# Variation in the post‐mating fitness landscape in fruit flies

**DOI:** 10.1111/jeb.13090

**Published:** 2017-05-12

**Authors:** C. Fricke, T. Chapman

**Affiliations:** ^1^ School of Biological Sciences University of East Anglia Norwich Research Park Norwich UK; ^2^ Institute for Evolution and Biodiversity University of Muenster Muenster Germany

**Keywords:** *Drosophila melanogaster*, selection analysis, selection gradient, sex peptide, sexual conflict, thin‐plate spline

## Abstract

Sperm competition is pervasive and fundamental to determining a male's overall fitness. Sperm traits and seminal fluid proteins (Sfps) are key factors. However, studies of sperm competition may often exclude females that fail to remate during a defined period. Hence, the resulting data sets contain fewer data from the potentially fittest males that have most success in preventing female remating. It is also important to consider a male's reproductive success before entering sperm competition, which is a major contributor to fitness. The exclusion of these data can both hinder our understanding of the complete fitness landscapes of competing males and lessen our ability to assess the contribution of different determinants of reproductive success to male fitness. We addressed this here, using the *Drosophila melanogaster* model system*,* by (i) capturing a comprehensive range of intermating intervals that define the fitness of interacting wild‐type males and (ii) analysing outcomes of sperm competition using selection analyses. We conducted additional tests using males lacking the sex peptide (SP) ejaculate component vs. genetically matched (*SP* ^*+*^) controls. This allowed us to assess the comprehensive fitness effects of this important Sfp on sperm competition. The results showed a signature of positive, linear selection in wild‐type and *SP* ^*+*^ control males on the length of the intermating interval and on male sperm competition defence. However, the fitness surface for males lacking SP was distinct, with local fitness peaks depending on contrasting combinations of remating intervals and offspring numbers. The results suggest that there are alternative routes to success in sperm competition and provide an explanation for the maintenance of variation in sperm competition traits.

## Introduction

Post‐copulatory male–male contests in the form of sperm competition were first described by Parker (1970) and, since then, huge research effort has been dedicated to understanding and identifying the underlying mechanisms involved (reviewed in Simmons, 2001). Studies conducted on diverse vertebrates, and particularly controlled laboratory experiments in invertebrate systems, have highlighted the importance to success in sperm competition of the timing of matings, reproductive trait morphology, differential sperm quality and the actions of seminal fluid proteins (Simmons, 2001; Pizzari & Parker, 2009; Sirot *et al*., 2014). The results show that interactions between the ejaculates of different males inside females are extremely common and represent an important arena for determining the fitness of interacting males and females.

In insects, last male sperm precedence dominates, and data from *Drosophila melanogaster* suggest that the degree of second male sperm precedence is associated with male lifetime reproductive success (Fricke *et al*., 2010). Here, sperm competition occurs after a female has remated and both first and second male ejaculates are simultaneously present to compete for fertilizations. Male sperm ‘offence’ is one of two contrasting roles males can adopt and occurs when a male encounters a mated female and hence has to gain a remating in order to dominate the subsequent fertilization set and outcompete the first mate. This measure is often quantified as P2 (the proportion of second male paternity). In contrast, when a male is the first to mate, his reproductive success is increased by defending his ejaculate against competition with, or usurpation by, ejaculates of subsequent mating males (sperm ‘defence’). This is often measured in double‐mating assays as P1 or the proportion of paternity gained by the first male after a second mating occurred. One potentially successful male strategy can be to delay the onset of remating to increase the exclusive usage of first male sperm for fertilizations before entering sperm competition. Supporting this, a longer interval between two matings often results in higher offspring productivity for the first male (e.g. Snook, 1998). However, we know little about the contribution of the number of offspring produced during the intermating period sired by the first male to fitness, and the sperm competition dynamics that ensue.

It has long been assumed that females would only remate when their sperm stores and productivity had declined (Gromko *et al*., 1984). Reduced sperm representation of the first male would then lead to a higher paternity share for the second male. In line with this, there is an overall trend for second male paternity share to increase with extended intermating interval (Simmons, 2001). However, this effect depends on the timing of remating and the dynamics of egg laying. For example, in the seed beetle *Callosobruchus maculatus*, manipulation of oviposition opportunities results in the second male gaining higher paternity scores (Eady *et al*., 2004). Without manipulation, the length of the remating interval has no effect on second male paternity share (Eady *et al*., 2004). However, there are notable exceptions, for example in the solitary wasp *Aphytis melinus,* extensions of the time to remating, through the agency of mate guarding by the first male, significantly reduces the second male's paternity gain (Allen *et al*., 1994). Similarly, longer remating intervals decrease second male paternity share in the water strider *Gerris lacustris,* (Danielsson & Askenmo, 1999) or have no effect on paternity, as in the flour beetle *Tribolium castaneum* (Bernasconi *et al*., 2006) or *Drosophila montana* (Aspi, 1992). The mechanisms underlying these patterns are not clear, but sperm storage dynamics (Danielsson & Askenmo, 1999; Eady *et al*., 2004) and female sperm storage organ morphology (Bernasconi *et al*., 2006) seem likely explanations. All these studies either measured offspring production during the intermating interval or paternity share of the second male, but none measured both traits simultaneously. Our main aim here was to capture pre‐ and post‐remating data, in order to better describe the reproductive potential of males in the first male role and how their productivity was shaped first by the period of exclusive access to females’ eggs and then by the paternity gained after remating.

We used the *Drosophila melanogaster* fruit fly model system both to capitalize on genetic tools and on the wealth of relevant background knowledge. For example, at remating, sperm from the current mate physically displaces resident sperm (Manier *et al*., 2010), with longer, slower sperm being more likely to remain in the fertilization set (Lüpold *et al*., 2012). Following mating, excess sperm are often ejected by the female (Snook & Hosken, 2004; Manier *et al*., 2010) and a male's subsequent success in sperm competition is dependent on the numerical representation of his sperm in the seminal receptacle (Lüpold *et al*., 2012).

Nonsperm components, particularly those seminal fluid proteins and peptides (Sfps) produced in the accessory glands of the male reproductive tract, are also key (Chapman, 2001; Fiumera *et al*., 2005, 2007). For example, although sperm traits aid males in remaining in competition in the fertilization set (Manier *et al*., 2010; Lüpold *et al*., 2012), Sfps such as Acp36DE are involved in ensuring the efficient storage of sperm (Neubaum & Wolfner, 1999). Other Sfps regulate the efficient retention (Acp29AB, Wong *et al*., 2008) or release (sex peptide (SP) or Acp70AA, Avila *et al*., 2010) of sperm from storage, which can alter the outcome of sperm competition (Chapman *et al*., 2000; Wong *et al*., 2008; Fricke *et al*., 2009; Avila *et al*., 2010). A number of other Sfps are also important determinants of sperm competitive success for males in both the first and second mating roles. A key aspect of sperm defence is a male's ability to delay his ejaculate entering into sperm competition by extending the period in which females are unwilling to remate. The length of the intermating interval is rarely investigated in detail in standard experimental set‐ups. In *D. melanogaster,* female willingness to remate is suppressed by transfer of SP (Chapman *et al*., 2003; Liu & Kubli, 2003; Smith *et al*., 2009). This delay benefits males (Fricke *et al*., 2009) and variation in SP expression is associated with the length of time to remating (Smith *et al*., 2009). Apart from an effect on remating latency, SP also increases female egg output (Chapman *et al*., 2003; Liu & Kubli, 2003) and regulates sperm release from female storage organs (Avila *et al*., 2010), thus altering sperm offence dynamics (Fricke *et al*., 2009; Avila *et al*., 2010).

Collectively, these findings show that many traits affect male sperm competitive success. A further characteristic of such traits is that they exhibit wide phenotypic and genetic variability (Fiumera *et al*., 2005, 2007; Snook *et al*., 2010; Lüpold *et al*., 2012). This is important in the context of the existence of complex nontransitive sperm competition dynamics (Clark *et al*., 2000; Zhang *et al*., 2013), which may contribute to the maintenance of genetic variation. Hence, there may be different routes to success via different male roles, or complex dynamics and/or unknown underlying trade‐offs between different determinants of sperm competitive ability. A potential problem in making progress in this context is that in order to understand the complexity, it is important to capture the full range of post‐mating interactions. We addressed this issue here by measuring the outcome from a more comprehensive and unmanipulated range of intermating intervals.

To do this, we aimed to use a design to address three potential concerns with sperm competition studies in *D. melanogaster* in which sexually mature females are mated to one male and then 24–72 h later to a second male. (i) First mating males who are good defenders and prevent females from remating in the time window set by the researcher are likely to be discarded from the experiment, as only double‐mated females are retained. These exclusions may penalize the most successful males, that is those that are most effective at preventing females from remating. (ii) The use of specified longer remating interval time points (e.g. 72 h) may result in high remating rates overall, but lump together the best and the poorest sperm defenders in one grouping. iii) Some studies have given females remating opportunities over several days without direct observations. These will lack detailed knowledge of when or how often females remated and include variable numbers of good and poor sperm defenders. The overall effect of (i–iii) is to obscure the precise sperm competition dynamics. It is also important to understand the whole landscape of the interactions between males during sperm competition to identify and accurately quantify potential trade‐offs. We gained a more comprehensive understanding of the fitness determinants of first mating males, by examining the relationship between the intermating interval and the number of offspring gained during this period. We then tested how these two variables affected sperm competition dynamics by scoring male success in sperm defence. We did this for wild‐type males and then for males lacking male ejaculate sex peptide (SP), in order to understand the impact of this key seminal fluid protein on sperm competition dynamics across the whole range of remating intervals. We then compared fitness landscapes for wild‐type, sex peptide‐lacking and SP^+^ control males and estimated selection gradients for the intermating interval and offspring number in relation to their impact on male fitness.

## Materials and methods

### Culturing methods

Dahomey wild‐type flies for these experiments were from an outbred population collected in the 1970s in Benin, Africa, and maintained in the laboratory since then. The populations were maintained at 25 °C and ~60% RH and a 12‐h:12‐h light: dark cycle in cages held at large population size with overlapping generations. All stocks were maintained on standard sugar–yeast (SY) food (100 g brewer's yeast, 50 g sucrose, 15 g agar, 30 mL Nipagin (10% w/v solution), 3 mL propionic acid, 1 L water). To collect eggs for the experiments, females were allowed to oviposit on an agar–grape juice plate (50 g agar, 600 mL red grape juice, 42.5 mL Nipagin (10% w/v solution), 1.1 L water) containing a smear of yeast paste. The next day, 100 first‐instar larvae were transferred to a glass vial (75 mm height × 25 mm diameter) containing 7 mL of SY food with the addition of live yeast granules. This standard density rearing reduced environmentally determined variation in adult body size, and any residual variation has little effect on mating trait expression (Edward & Chapman, 2012). Eclosing adults were collected under ice anaesthesia, sorted by sex and held in single sex groups of ten. We allowed these adults to mature for 2–3 days before use in the experiment.

### Fitness landscape of wild‐type males in sperm competition

On the first day of the experiment, we took 100 Dahomey wild‐type males and mated them individually to a virgin wild‐type female each. We observed pairs continuously, recorded the beginning and end of each mating and discarded pairs if they did not mate at all within 2 h. Immediately after a successful mating ended, the male was removed and replaced by a wild‐type competitor male carrying the dominant *Stubble* (*Sb*) mutation as a marker phenotype. Marker males were matched to the wild‐type genetic background by backcrossing *Birmingham;Sb[1]/TM6* (Bloomington Drosophila Stock Centre #2539) for six generations into the Dahomey genotype, to generate *Dah;Sb[1]*. After the initial matings, we continued to watch pairs continuously to record rematings. If a remating did not happen within 6 h of observation, pairs were separated and females were held singly until the next day when they were provided again with a competitor male for 6 h and again observed continuously. This was repeated every day until remating occurred for all of the once‐mated females. The latency and duration of mating were recorded for the first as well as the second mating. The intermating interval was calculated as the time between the start times of the two successive matings. After a successful second mating, the male was removed and the female transferred to a fresh vial to oviposit. Females were then transferred into new oviposition vials daily for 4 days before being discarded. All vacated vials, as well as the first set of vials from before the second mating, were then incubated for 12 days to allow the offspring to emerge. We counted the total number of offspring, including those produced during the intermating interval before the second mating. Offspring produced after the second mating were counted and scored for the *Sb* or non‐*Sb* phenotype to assign paternity. Thus, for each wild‐type male, we gained data on the length of the intermating interval, the number of offspring gained during this period, sperm competitive success and total offspring production.

### Fitness landscape of SP‐lacking and SP‐transferring males in sperm competition

We repeated the above experiment using as the first male a SP‐lacking (*SP* ^*0*^) or a SP‐transferring (*SP* ^*+*^) control male and *Dah Sb[1]* males as the second mates, as before. The sample size was 100 males of each genotype initially mated to a virgin Dahomey wild‐type female. *SP* ^*0*^ and *SP* ^*+*^ control males were generated by crossing virgin *Δ130/TM3, Sb, ry* females to *SP* ^*0*^ */TM3, Sb, ry* or *SP* ^*0*^ *,SP* ^*+*^ */TM3, Sb, ry* males, respectively. *Δ130/SP* ^*0*^ male offspring do not produce and transfer SP, and *Δ130/SP* ^*0*^ *,SP* ^*+*^ male offspring were SP‐transferring, genetically matched controls (Liu & Kubli, 2003). All lines used to generate the *SP* ^*+*^ and *SP* ^*0*^ males had been backcrossed into the Dahomey wild‐type background. *Δ130/TM3, Sb, ry* was backcrossed for three generations and chromosomes 1, 2 and 4 of the *SP* ^*0*^ */TM3, Sb, ry* and *SP* ^*0*^ *,SP* ^*+*^ */TM3, Sb, ry* stocks for four generations. The sperm competition experiment was conducted exactly as described above with the exception that after the second mating, we only allowed females to lay eggs over two 24‐h periods instead of four.

### Statistical analysis

Analyses were mainly performed using R v.3.2.1 (R Development Core Team 2015) and RStudio v.0.98.1103. Correlations were performed using SPSS v20. We present summary data as means ± SE throughout. We performed selection analyses to test for linear and nonlinear selection pressures on male‐induced latency to remating and male sperm defence ability. Prior to these analyses, we calculated male relative fitness (w) for the first male to mate. First, we summed the total number of offspring produced before and after remating per male and then for each individual male, we calculated his relative fitness compared to his treatment group by dividing the total number of offspring produced by the mean (Lande & Arnold, 1983). When performing calculations for the *SP* ^*0*^ and the *SP* ^*+*^ control males, each treatment was standardized separately by its treatment mean. Latency to remating and male competitive success, measured as the proportion of offspring gained by the first male (number of first male offspring after remating divided by the total number of offspring produced after remating), were both z‐score‐standardized to units of standard deviation with a mean of 0. Using male relative fitness as the response variable and these standardized data for latency to remating and the proportion of first male offspring after remating as the explanatory variables, we performed multivariate first‐order and second‐order polynomial regressions to test for linear and nonlinear selection (Lande & Arnold, 1983; Brodie *et al*., 1995). We compared models of varying complexity and inspected the diagnostic plots to select the best error distribution. We report the results as partial F‐tests to first test whether linear selection and nonlinear selection were acting, before proceeding to estimate the mode and strength of selection on latency to remating and first male sperm defence ability. First‐order polynomial regression provided the selection gradient *β*, which was given by the partial regression coefficients from the multivariate regression. The cross‐product (*γ*_*ij*_) and quadratic regression coefficient (*γ*_*ii*_ *)* for our traits of interest, as estimated in a second‐order polynomial, describe the curvature of the selection surface (Lande & Arnold, 1983). These coefficients were used to build a gamma matrix and perform canonical rotation to account for the observation that *γ* often underestimates the strength of nonlinear selection (because cross‐products can represent correlational selection between traits (Blows & Brooks, 2003)). Canonical rotation controls for correlation between traits by eliminating the cross‐products and estimating the major axes of the response surface. Each new eigenvector (m_i_) represents one major axis and the contribution from the variables tested (here latency to remating and male P1 success) on a new composite trait is calculated (Phillips & Arnold, 1989; Blows & Brooks, 2003). Prior to canonical rotation, the quadratic coefficients in the gamma matrix were doubled to correctly estimate the mode of selection (Stinchcombe *et al*., 2008). The contribution of the original traits to the major axes, as revealed by canonical rotation, was then determined. These new variables were then placed back into a second‐order polynomial regression, with the quadratic terms in the model representing the significance of nonlinear selection (see Blows *et al*., 2003).

We also compared the selection surfaces for the *SP* ^*0*^/*SP* ^*+*^ experiment. Our analysis contained two traits of interest, and we therefore followed the steps outlined in Rundle *et al*. (2005). We tested separately for differences between treatments in linear and nonlinear selection on latency to remating and first male sperm defence ability. To test for differences in linear selection between first matings with *SP* ^*0*^/*SP* ^*+*^ on latency to remating or male sperm defence ability, we extended the model to include the interaction terms between treatment and our two traits. We then used a single partial F‐test and compared the full model including the two interaction terms with a model lacking both terms. Comparing the curvature of nonlinear selection, we added all possible two‐way interactions as well as all possible three‐way interactions between the treatment and the quadratic and cross‐products. We then compared the full model with the reduced model from which all three‐way interactions had been removed, and reported the results from the partial F‐test.

To display the data, we either used plot.gam (2D figures) from the mgcv package (Wood, 2015) or the Tps command (3D figures) from the fields package (Nychka *et al*., 2015). The mgcv package facilitates the use of generalized additive models in which a nonparametric smoother is fitted to the data (Crawley, 2007). In this, the fitness function corresponds to a cubic spline and portrays the relationship between fitness and trait values for individuals (Schluter, 1988). The graphs representing fitness functions as cubic splines are from models containing both traits of interest, a Gaussian error distribution and smoothing parameters fitted as the value that minimizes the generalized cross‐validation (GCV) score (Schluter, 1988). The Tps command in the fields package similarly uses the GCV score to fit a thin‐plate spline regression to portray the relationship between fitness, latency to remating and first male sperm defence ability in a contour or 3D plot.

## Results

### Fitness landscape of wild‐type males in sperm competition

**Figure 1:**
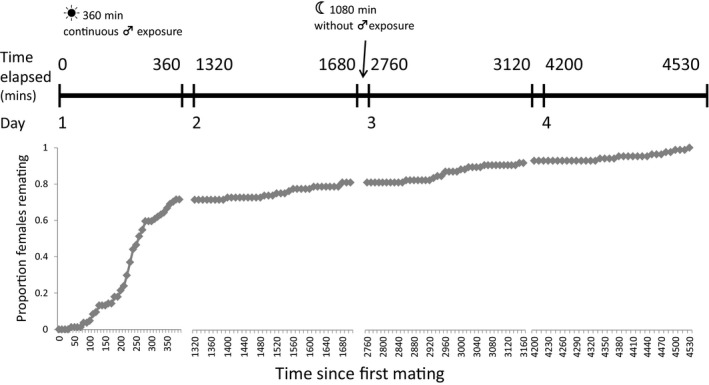
Cumulative remating rate against time (in minutes) for the wild‐type male experiment. 96 Dahomey wild‐type females were mated as virgins to a Dahomey wild‐type male and then exposed to a second wild‐type *Dah;Sb[1]* male for 6 h each day for a maximum of 4 days until a second mating occurred.

**Figure 2:**
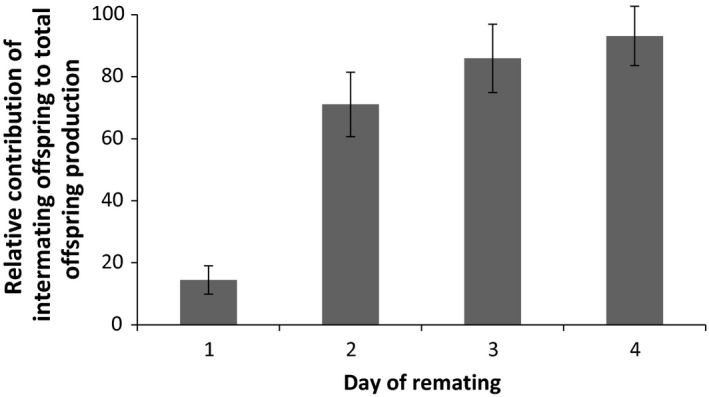
Relative proportion of offspring produced in the intermating interval out of the total number of offspring produced, against day of remating for the wild‐type experiment. The number of offspring from the intermating period represented a successively larger fraction of overall offspring production as males induced longer remating intervals (increasing day of remating).

A total of 96 virgin females successfully mated a first time, and of these, 62.5% remated within 6 h immediately following the first mating. Of the remaining females, 52.7% remated during the 6‐h observation period the second day, whereas a minority of females (*n* = 7) were not willing to remate until day 4 (Fig. 1). In terms of the simple binary relationships between traits, we observed that males able to elicit a longer refractory period produced significantly more offspring in the intermating period (Spearman's rho = 0.884, *P *<* *0.001; Fig. S1a), but fathered fewer offspring after remating (*r *=* *−0.437, *P *<* *0.001). Thus, males that elicited a long refractory period were less successful when entering sperm competition (*r *=* *−0.375, *P *<* *0.001; Fig. S1b). There was also a significant negative relationship between the number of offspring produced before and after remating (*r *=* *−0.339, *P *=* *0.001, Fig. S2). The length of the remating interval was an important determinant of male fitness as it was significantly positively correlated with total focal offspring production (*r *=* *0.408, *P *<* *0.01). The number of offspring produced during the intermating period contributed a larger fraction of overall offspring production for males inducing longer remating intervals (Fig. 2).

**Figure 3:**
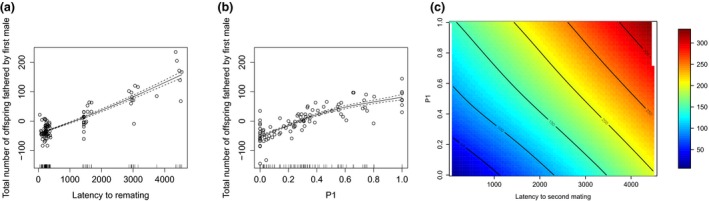
Wild‐type male fitness, latency to second mating and P1. Panels (a) and (b) show cubic splines of the relationship between male fitness and (a) intermating interval (latency to second mating), or (b) male sperm defence ability (P1). Panel (c) shows the relationship between fitness, intermating interval (latency to second mating) (*x*‐axis, minutes) and sperm defence ability (*y*‐axis, P1). The colours show the potential fitness gain resulting from the different combinations of remating interval and P1. Male fitness is the total number of offspring fathered by the first male before and after remating. Latency to remating was measured as the time between a female's first mating and second mating, in minutes. After the first successful mating, the male was immediately replaced with a new male and pairs were continuously observed for 6 h daily until a remating occurred. After remating, offspring were collected over the next 4 days and the proportion offspring fathered by the first male was scored. Hatched lines represent ± SE.

The selection analyses tested for the strength and form of selection acting on male‐induced latency to female remating and sperm competitive success. The results showed that the first‐order polynomial regression, which included standardized latency to remating and P1, provided a good model fit and significant explanation of the variation in male fitness (*F*_2,92_ = 159.28, *P *<* *0.001, *r* ^2^ = 0.78). Removing these two traits in turn from the full model with Gaussian errors in an analysis of deviance showed that both traits contributed significantly to variation in fitness (partial F‐tests; latency to remating: *F*_1,93_ = 243.12, *P *<* *0.001; male sperm defence: *F*_1,93_ = 163.48, *P *<* *0.001). Both variables were under positive linear selection, with slightly stronger selection acting on latency to remating (Table S1). Fitness increased in a monotonic fashion as male‐induced latency to remating increased (Fig. 3a), whereas the relationship of fitness with increasing P1 reached a plateau and thereafter seemed to give diminishing returns (Fig. 3b). Extending the model to include the square and cross‐products revealed evidence for nonlinear selection also acting on the two traits (*F*_3,89_ = 3.59, *P *=* *0.017). The negative quadratic coefficient for male sperm defence ability (P1) indicated that there was a convex curvature of the selection surface for this trait, with multiple fitness peaks (Table S1).

The canonical rotation confirmed that the major curvature of the fitness surface was caused by variation in male sperm defence ability and less so by male‐induced latency (Table S1). This curvature was significant as shown by the finding that exclusion of the two cross‐product terms significantly reduced the fit of the model (*F*_2,92_ = 5.45, *P *=* *0.006). However, it was the new variable m_2_ that demonstrated significant stabilizing selection (*P* < 0.01, Table S1) and it mainly represented the contribution of P1. The value for m_1_ was marginally nonsignificant (*P *=* *0.053) with a major contribution from male‐induced latency to remating, indicating that the induction of longer latency was under strong selection (Table S1). Despite its significant relationship with fitness, the curvature was fairly modest (Fig. 3c, see Fig. S3 for a 3D representation).

### Fitness landscape of SP‐lacking and SP‐transferring control males in sperm competition

**Figure 4:**
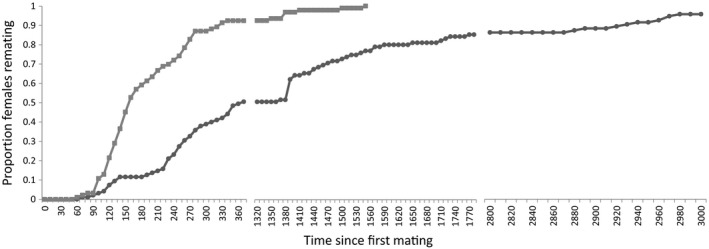
Cumulative remating rate against time (in minutes) in the SP male experiment. 94 wild‐type Dahomey females were mated as virgins to males lacking sex peptide (*SP* ^*0*^, light grey with squares) and 96 to a sex peptide‐transferring control male (*SP* ^*+*^, dark grey with dots). Both sets of females were then exposed to a wild‐type *Dah;Sb[1]* male for 6 h each day until a second mating occurred.

As expected, SP transfer was a key component in determining the length of male‐induced remating latency and was also key to the extent of overall male fitness benefits. 94% of virgin females mated with a SP‐lacking male, and of these, 92.5% remated within 6 h and all the remaining females remated the following day. In contrast, 97% of virgin females accepted a first mating with a *SP* ^*+*^ control male and of these females only 50.5% remated within 6 h. On the second day, 70.2% of the remaining females remated, whereas three females did not remate until day 4 of the experiment and one female did not remate at all (Fig. 4). Females mated to *SP* ^*+*^ control males remated on average after 1125.3 ± 111.8 min, whereas females not receiving SP during their first mating remated after only 276.7 ± 35.3 min. A Mann–Whitney *U*‐test revealed that the distribution of latency to remating was significantly different for the *SP* ^*+*^ and *SP* ^*0*^ treatments (*P *<* *0.001).

**Figure 5:**
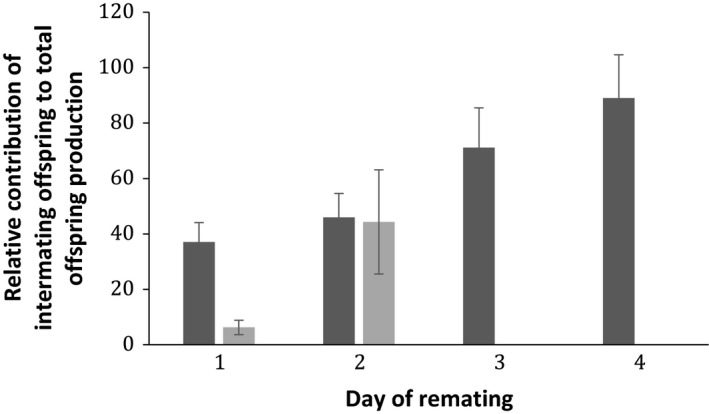
For the comparison of SP‐lacking (*SP* ^*0*^, light grey) with SP‐transferring control males (*SP* ^*+*^, dark grey), the relative proportion of the number of offspring produced in the intermating interval out of the total number of offspring produced, against day of remating, is depicted. The number of offspring from the intermating period represented a successively larger fraction of overall offspring production as males induced longer remating intervals (increasing day of remating).

In both *SP* ^*+*^ and *SP* ^*0*^ treatments, males benefitted significantly from a longer intermating interval (positively correlated with the number of offspring; *SP* ^*0*^: Spearman's rho = 0.601, *P *<* *0.01; *SP* ^*+*^: *r *=* *0.785, *P *<* *0.01). However, the fitness pay‐off was much greater for the *SP* ^+^ control males (Fig. 5 and S4). On average, *SP* ^*0*^ males produced 6.1 ± 1.6 offspring during the intermating interval, whereas *SP* ^*+*^ control males had 43.1 ± 6.0 offspring *(t*_107.4_ = 5.95, *P *<* *0.0001). The length of the intermating period correlated positively with the number of offspring produced after a second mating for *SP* ^*+*^ control males (*r *=* *0.396, *P *<* *0.01, Fig. 5) and with the proportion of paternity gained (P1: *r *=* *0.460, *P *<* *0.01). In *SP* ^*0*^ males, the overall shorter intermating interval reduced the magnitude of these potential fitness benefits, as indicated by the nonsignificant relationships between fitness and offspring numbers after remating (*r *=* *0.130, *P *=* *0.216) or P1 (*r *=* *0.145, *P *=* *0.169). Even though *SP* ^*0*^ males gained more offspring after remating in absolute numbers (*SP* ^*0*^ = 37.4 ± 3.6; SP^+^ = 23.6 ± 3.6, *t*_185_ = 2.73, *P *=* *0.007), this was not enough to cancel out the loss of progeny during the intermating period, as shown by an overall higher reproductive success for control males (*SP* ^*0*^ = 43.5 ± 4.0; *SP* ^*+*^ = 66.6 ± 8.0 offspring in total; *t*_139_ = 2.59, *P *=* *0.011).

**Figure 6:**
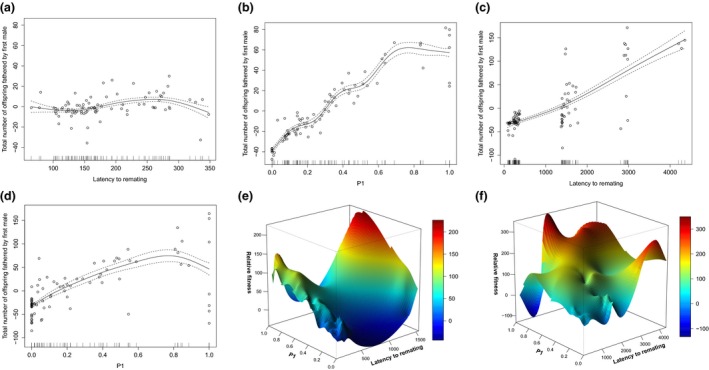
*SP* ^*+*^ and *SP* ^*0*^ male fitness, latency to second mating and P1. Cubic splines to illustrate the relationship between fitness and male‐induced latency to remating (a) + (c) and male sperm defence (P1) success (b) + (d) for *SP* ^*0*^ (a, b) and *SP* ^*+*^ (c, d) males. Note the different scales on the fitness ordinates for the two male genotypes. Data points for seven females (of 94) remating on day two in the *SP* ^*0*^ treatment were excluded as they exerted undue leverage and forced the model to define an area of badly represented parameter space, with a large gap to the remaining 87 data points. Similarly, one data point for the *SP* ^*+*^ treatment was excluded, as the female did not remate within the 4 day window. 3D plots of the fitness landscape for (e) SP‐lacking (*SP* ^*0*^) and (f) SP‐transferring, genetically matched control (*SP* ^*+*^) males. Displayed is the combined impact of the male‐induced length of the intermating interval and his sperm defence (P1) success on fitness. Fitness is measured as the total number of offspring produced by the focal male and is shown on the z‐axis. The height and colour of the landscape display the potential fitness gain resulting from the different combination of intermating interval and P1. The ruggedness in the surface for *SP* ^*+*^ control males for remating intervals longer than 2000 min should be interpreted with caution, as this part of the landscape was based on few data points (90% of females remated by day 3).

Selection analysis revealed evidence for significant linear selection (deviance = 107.68, *F*_2,183_ = 128.00, *P *<* *0.001; all models were generalized linear models fitted with a quasipoisson error distribution). Both male‐induced latency to remating (deviance = 27.67, *F*_1,184_ = 65.77, *P *<* *0.001) and male sperm defence ability (deviance = 58.98, *F*_1,184_ = 140.21, *P *<* *0.001) were under directional selection (Table S2). However, there was a clear difference for *SP* ^*+*^ and *SP* ^*0*^ males (deviance = 12.65, *F*_2,181_ = 18.18, *P *<* *0.001). The length of the intermating interval showed a loose relationship with fitness for the *SP* ^*0*^ males, in contrast to a strong contribution of success in sperm defence to overall fitness (Fig. 6a, b). This pattern was reversed for the control males, as there was a strong positive relationship between fitness and latency to remating and a monotonic increase with increasing P1 success (Fig. 6c, d). Fitness pay‐offs overall were higher for *SP* ^*+*^ than for *SP* ^*0*^ males.

There was also evidence for nonlinear selection shaping the relationship between fitness, the length of the remating interval and first male sperm defence (deviance = 37.48, *F*_3,180_ = 49.08, *P *<* *0.001). However, there was no significant difference overall in the individual fitness surfaces for the two male genotypes (i.e. *SP* ^*0*^ vs. *SP* ^*+*^ control; deviance = 1.06, *F*_3,175_ = 1.68, *P *=* *0.17, Fig. 6e,f). The fitness surface for *SP* ^*0*^ males had a clear saddle shape with lowest fitness returns at intermediate lengths of the intermating interval and two peaks at the two extreme ends. Fitness peaks at these two extremes occurred when combined with males gaining high paternity shares (Fig. 6e). The fitness surface for the *SP* ^*+*^ males was more rugged with highest returns at long intermating intervals. However, particularly for the later latencies, the surface needs to be interpreted with caution. Most females had remated by day 3 (~90% see Fig. 4), and hence, this part of the fitness landscape is based on few data. However, we did make use of all available replicates to strengthen our estimate when calculating the shape of the fitness surface. Local fitness peaks depended on sperm competition outcomes with one optimum each towards the two extremes of no or complete paternity share (Fig. 6f). These results should also be interpreted with caution, as a larger sample size might produce smoother fitness surfaces. However, we also note that a similar sample produced a smooth fitness surface in the wild‐type male experiment.

To further explore these patterns, we performed canonical rotation separately for the *SP* ^*0*^ and *SP* ^*+*^ male treatments, and used the combined table including data for both male genotypes in the analysis. Excluding both squared products from the regression significantly reduced the fit of the model (*F*_2,183_ = 19.98, *P *<* *0.001). Both m_1_ and m_2_ significantly explained curvature in the fitness surface for both male treatments. As for the wild‐type male analysis, the new vector m_1_ mainly represented the length of the intermating interval and m_2_ mainly success in male sperm defence (Table S2). However, the two traits displayed small shifts and reversal in trait combination in their loadings on the new axes for *SP* ^*0*^ vs. *SP* ^*+*^ males (Table S2; Fig. S5a,b). The results for the *SP* ^*+*^ treatment were similar to the wild‐type males, with latency to remating having a strong negative loading and male sperm defence a minor positive loading on m_1_ and both a positive loading on m_2_ (Fig. 5a). In the *SP* ^*0*^ treatment, both traits had a negative loading on m_1_ and also latency to remating loaded negatively on m_2_ (Table S2; Fig. 5b). Hence, the selection surfaces differed from each other for the two male genotypes, with different trait combinations causing curvatures in those surfaces.

## Discussion

We estimated the natural length of the intermating interval in twice‐mated females and found that about half of the females remated within 6 h after their first mating. Males that induced longer intervals gained significant fitness benefits by increasing their reproductive output and delaying the onset of sperm competition. The length of the time until remating also affected sperm competition dynamics, and we confirmed that the transfer of ejaculatory sex peptide (SP) was key to these processes.

A large fraction of females remated shortly after a first mating. Receipt of SP significantly affected these dynamics, as 90% of females not receiving SP remated within 6 h after a first mating, whereas only 50–60% of females did so when receiving SP. SP seems to require some time to exert its effect on female remating behaviour, and during this time window, when the response is still developing, early rematings may be frequent. This means that the method used to measure remating can affect the outcome observed. Our results are in contrast to previous work that reported few rematings (<10%) occurring 4 h after a first mating to *SP* ^*0*^ males (Liu & Kubli, 2003; Peng *et al*., 2005), but in line with other studies showing high early rematings (>60%) (Van Vianen & Bijlsma, 1993; Bretman *et al*., 2010; Smith *et al*., 2017). The length of female remating rate is determined by receipt of SP but also has a heritable basis (Sgro *et al*., 1998; Lüpold *et al*., 2013). Hence, differences between female genotypes could partly explain the inconsistent results. Our continuous exposure of females to second mating males, and the resulting high frequency of rapid rematings, could also be a result of high male activity and courtship (Boulton & Shuker, 2016).

The rapid rematings in the *SP* ^*0*^ treatment often occurred before any offspring from the first male were produced. Our evidence suggests that this high incidence was not due to pseudocopulations, as (i) all matings exceeded the threshold for sperm transfer of >5 min (Gilchrist & Partridge, 2000), (ii) females mated to *SP* ^*0*^ males are reported to have equal numbers of sperm in storage in comparison with controls (Avila *et al*., 2010), and (iii) in our previous work, we rarely observed infertile pairings from *SP* ^*0*^ matings (2/19 3‐day‐old females mated to *SP* ^*0*^ males produced no offspring, vs. 1/20 controls (Fricke *et al*., 2013)). Instead, the data highlight the importance of the oviposition‐enhancing effect of SP (Chapman *et al*., 2003; Liu & Kubli, 2003) and that male stimulation of female oviposition rate was key to male fitness gains during the intermating interval.

Our data show that female early remating occurs and can have profound effects on male reproductive success – delaying remating provided first males with large fitness benefits and this trait was under strong directional selection. The length of the intermating interval also impacts upon the outcome of sperm competition (Lüpold *et al*., 2013) as it can affect the number of sperm remaining in the fertilization set (Manier *et al*., 2010; Lüpold *et al*., 2012). In line with this idea, we found a negative correlation between length of the intermating interval and male P1 success (*r *=* *−0.313, *P *=* *0.002). In contrast, Fiumera *et al*. (2007), using 96 chromosome 3 substitution lines, allowed rematings after 48 h and instead reported a positive correlation between intermating interval fecundity and male sperm defence and offence success and strong variation in P1 success among lines. A similar pattern of a longer remating interval decreasing second male paternity has been reported in the water strider *G. lacustris* (Danielsson & Askenmo, 1999) and the solitary wasp *A. melinus* (Allen *et al*., 1994). Differences in the outcomes of how remating interval affects sperm competition could be due to male variance in fecundity‐enhancing efficiency or in the number of sperm transferred or stored. Under a scenario where males induce long remating intervals, differences in the ability to elicit female oviposition would affect not only the paternity gained before entering sperm competition but also the number of sperm remaining in the fertilization set, hence sperm defence ability.

Sperm competition dynamics may be very different in early rematings (Smith *et al*., 2017). Sperm competition is initiated in many existing studies after the sperm of the first male has already been stored and used for fertilization (e.g. Manier *et al*., 2010; Lüpold *et al*., 2012, 2013). Our work here captured sperm competition dynamics across an extended range covering the period before first male sperm is fully stored. This included the period during which first male sperm can be ejected and during which the first eggs transit the female reproductive tract. Males in our study could still gain a high proportion of fertilizations even if females remated within 6 h after the first mating. This indicates that sufficient sperm were still retained in the fertilization set. SP could be a mediator of these dynamics, with pleiotropic effects – benefitting male reproductive output after a single mating (Chapman *et al*., 2003; Liu & Kubli, 2003; Fricke *et al*., 2009) and regulating sperm release from storage (Avila *et al*., 2010). However, SP appears to play no role in the transit of sperm into storage (Avila *et al*., 2010). Instead, SP might protect sperm from being replaced after they successfully entered storage. Equal numbers of sperm from *SP* ^*0*^ and *SP* ^*+*^ males are found in storage shortly after mating, but 4 days after mating significantly more sperm from *SP* ^*0*^ males remains, in comparison with sperm from *SP* ^*+*^ males (Avila *et al*., 2010). This is consistent with the finding that particularly in the *SP* ^*0*^ male treatment, we observed high P1 values after early rematings, in which females were likely to retain many stored sperm. For the SP‐lacking males, we found a strong link between male sperm defence success (P1) and fitness, whereas the relationship between fitness and length of the intermating interval was flat. This impact of the length of the intermating interval and P1 on fitness shifted when males transferred SP, and the length of the intermating interval had a major effect on male fitness gains. Both SP‐transferring control and wild‐type males showed no covariation between sperm defence and remating inhibition. In contrast, in the *SP* ^*0*^ treatment, we found a significant positive signature of a correlational selection gradient between these two traits. This might be due to pleiotropic effects in the *SP* ^*0*^ males, where a lack of SP results in both early rematings and more sperm remaining in the fertilization set due to fewer sperm being released from storage (Avila *et al*., 2010).

In wild‐type and control males, the length of intermating interval and P1 both positively impacted on fitness and showed evidence of linear and nonlinear selection. One combination that led to maximum fitness was a long intermating interval and high P1 values. However, latency to remating strongly affected male fitness, and for male sperm defence, the fitness surface revealed some curvature with diminishing returns. There was little evidence that both traits jointly determined fitness or that there was a trade‐off, as the length of the intermating interval and male sperm defence ability had very distinct loadings on the new axes after canonical rotation, indicating that their effects were largely independent. Thus, although maximum gain was reached by combining high values for both traits, a loss in offspring production before engaging in sperm competition could not be rebalanced by high P1 values. An additional source contributing to male fitness was variation in female fecundity. Variation among males in the extent to which they can enhance female egg laying is expected to contribute to overall differences in female fecundity (Smith *et al*., 2009; Tennant *et al*., 2014) as well as female condition. However, how much variation in female fecundity contributes to male fitness might also depend on a male's genotype. SP‐lacking males induce lower rates of oviposition in females and, combined with quick rematings, male reproductive success is mostly explained by P1 and variation in female fecundity. In contrast, for SP‐transferring males, the length of the intermating interval and the male's fecundity‐enhancing ability contribute more strongly to reproductive success, and variation in female fecundity is expected to explain less of the variation in male fitness.

The relationship of male‐induced latency to remating and sperm defence success, with fitness was nearly a plane for wild‐type males, yet more rugged for the *SP* ^*+*^ control males. The differences might be due to variation in husbandry, such as lower‐density culture for the *SP* ^*+*^ in comparison with wild type maintained in cage cultures. The parental lines to generate the *SP* ^*0*^ and *SP* ^*+*^ males were back‐crossed into the genetic background of the Dahomey wild type, except for chromosome 3, on which the SP deletion, and a number of sperm competition genes, is present (Fiumera *et al*., 2007). It is possible that individual replicates with strong phenotypes could have had an above‐average influence on some extreme points, resulting in a rugged fitness surface. However, we think this less likely for the reason that the wild‐type population at similar sample size gave a smooth plane.

Even despite the differences in the shape of the fitness surfaces, both types of males shared a similar pattern, where fitness was maximized at long intermating intervals and towards high P1 values. This was in stark contrast to the fitness surface of *SP* ^*0*^ males, which showed a distinct saddle shape at intermediate values for both traits. In the *SP* ^*0*^ treatment, males with early rematings still gained fitness, particularly in combination with high sperm defence.

By observing the length of intermating intervals and measuring fecundity before and sperm competition dynamics after remating, we highlighted determinants of male reproductive success. This extended existing protocols to encompass shorter remating intervals and avoided minimizing the fitness of good sperm defenders. However, it may not fully reflect dynamics in nature. We restricted dynamics to one remating and, while this might be representative of the dynamics of triple matings (Morrow *et al*., 2005), extending the approach to more realistic scenarios to study more traits and hence study potential trade‐offs would be useful. In conclusion, we measured across the naturally occurring length of the intermating period and observed complexity in male fitness surfaces and revealed different routes to fitness maxima. Hence, the existence of such peaks shows evidence for the maintenance of genetic variation in traits related to sperm competition arising due to sexual competition and conflict (Hall *et al*., 2008, 2010).

## Conflict of interests

The authors declare that they have no conflict of interests.

## Supporting information


**Figure S1** For the wild type male experiment, the relationship between the length of the intermating interval (in minutes) and (a) the number of offspring gained by the first male to mate before remating occurred, (b) the proportion of offspring (P1) gained by the first male to mate.
**Figure S2** For the wild type male experiment, the number of offspring produced before and after remating.
**Figure S3** A 3D representation of the fitness surface for wild type experiment males for two post‐mating traits: male induced length of remating interval and sperm defence ability.
**Figure S4** The relationship between the length of the intermating interval and the number of offspring produced during this interval for (a) *SP* ^*0*^ males and (b) *SP* ^*+*^ control males.
**Figure S5** Fitness surfaces for *SP* ^*0*^ (a,c) and *SP* ^+^ (b,d) control males after canonical rotation, where axis M_1_ mainly represents the length of the remating interval and axis M_2_ a male's success in sperm defence.
**Table S1** Tests for directional selection on mating‐induced latency to remating and first male sperm defense ability in the wild type male experiment.
**Table S2** Tests for directional selection on mating‐induced latency to remating and first male sperm defense ability in the *SP* ^*0*^ and *SP* ^+^ control male experiment.
