# Supplementary material for: Variation in the post‐mating fitness landscape in fruit flies
Source: J Evol Biol. 2017 May 12;30(7):1250–61. doi: 10.1111/jeb.13090 (PMC5518202; doi:10.1111/jeb.13090)
Supplement: Supplementary file 1 — Figure S1 For the wild type male experiment, the relationship between the length of the intermating interval (in minutes) and (a) the number of offspring gained by the first male to mate before remating occurred, (b) the proportion of offspring (P1) gained by the first male to mate. Figure S2 For the wild type male experiment, the number of offspring produced before and after remating. Figure S3 A 3D representation of the fitness surface for wild type experiment males for two post‐mating traits: male induced length of remating interval and sperm defence ability. Figure S4 The relationship between the length of the intermating interval and the number of offspring produced during this interval for (a) SP 0 males and (b) SP + control males. Figure S5 Fitness surfaces for SP 0 (a,c) and SP + (b,d) control males after canonical rotation, where axis M1 mainly represents the length of the remating interval and axis M2 a male's success in sperm defence. Table S1 Tests for directional selection on mating‐induced latency to remating and first male sperm defense ability in the wild type male experiment. Table S2 Tests for directional selection on mating‐induced latency to remating and first male sperm defense ability in the SP 0 and SP + control male experiment. [file JEB-30-1250-s001.pdf]

Variation in the post-mating fitness landscape in fruitflies

Claudia Fricke[1,2] & Tracey Chapman[1,*]

[1] *School of Biological Sciences, University of East Anglia, Norwich Research Park, NR4 7TJ, UK*

[2] *Institute for Evolution and Biodiversity, University of Muenster, 48149 Muenster, Germany*

Supporting information

Table S1, S2

Figures S1-S5

Table S1. Tests for directional selection on mating-induced latency to remating and first male sperm defense ability in the wild type male experiment. (a) Vector of standardized directional selection gradients (β) and the matrix with standardized quadratic and correlational gradients (γ) and (b) the matrix of eigenvalues and eigenvectors after canonical rotation. Quadratic regression coefficients were doubled, to correctly estimate quadratic selection gradients (Stinchcombe *et al.,* 2008).

(a)	**β**	**γ**	
		**Latency**	**P1**
Latency to remating	0.534***	0.120	
P1	0.438***	-0.003	-0.188**
(b)	λ	Latency	P1
m_1	0.120	-1.000	0.010
m_2	-0.188*	0.010	1.000

* <0.01, ** <0.001, ***<0.0001

Table S2. Tests for directional selection on mating-induced latency to remating and first male sperm defense ability in the SP^0 and SP^+ control male experiment. (a) Vector of standardized directional selection gradients (β) and the matrix with standardized quadratic and correlational gradients (γ) and (b) after canonical rotation the matrix of eigenvalues and eigenvectors. Quadratic regression coefficients were doubled, to correctly estimate quadratic selection gradients (Stinchcombe *et al.,* 2008).

**Trait**	***SP⁰* Males**			***SP⁺* Males**		
(a)	**β**	**γ**		**β**	**γ**	
		**Latency**	**P1**		**Latency**	**P1**
Latency	0.263***	0.142		0.732***	0.264*	
P1	0.759***	0.080**	-0.362***	0.473***	-0.054	-0.538**
(b)	λ	Latency	P1	λ	Latency	P1
m_1	0.154*	-0.988	-0.153	0.268*	-0.998	0.067
m_2	-0.374***	-0.153	0.988	-0.542**	0.067	0.998

*<0.05, ** <0.001, ***<0.0001

Figure S1 For the wild type male experiment, the relationship between the length of the intermating interval (in mins) and (a) the number of offspring gained by the first male to mate before remating occurred, (b) the proportion of offspring (P1) gained by the first male to mate. The intermating interval was measured as time between the end of the first mating and the start of the second (first male, Dahomey wild-type; second, *Dah;Sb[1]*).

(a)

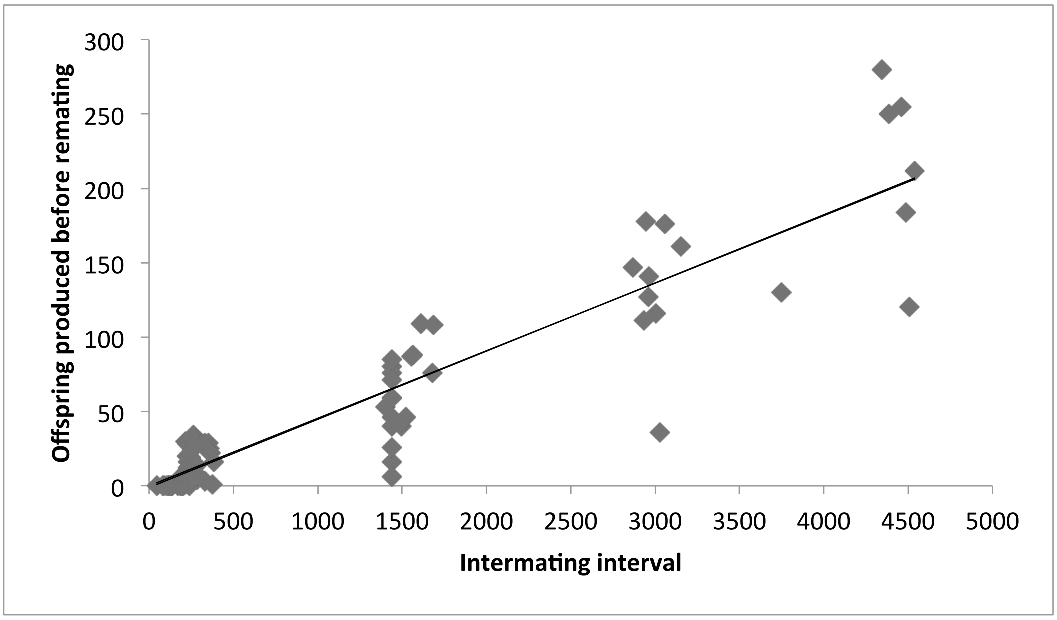

(b)

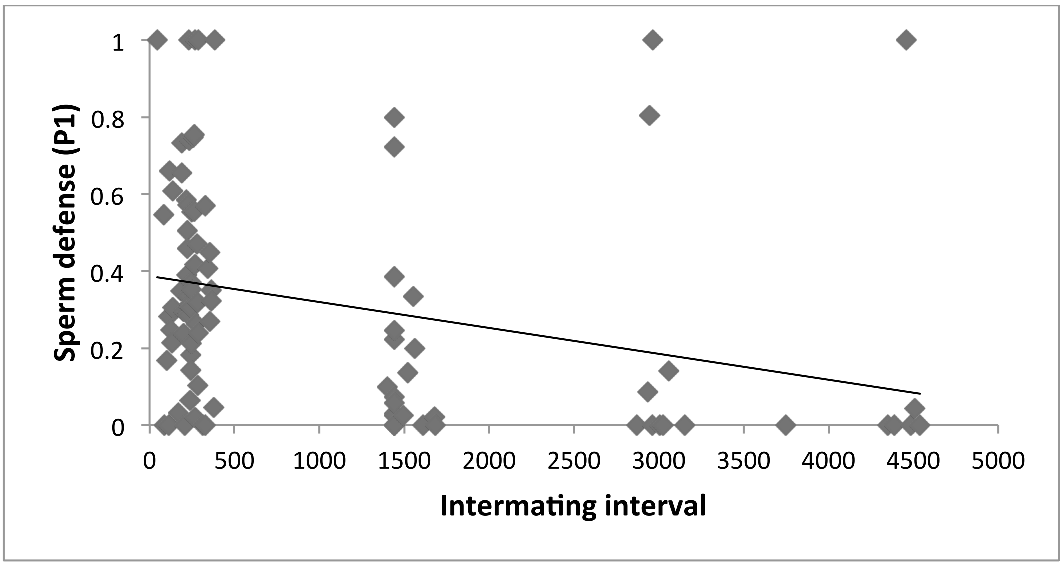

Figure S2 For the wild type male experiment, the number of offspring produced before and after remating.

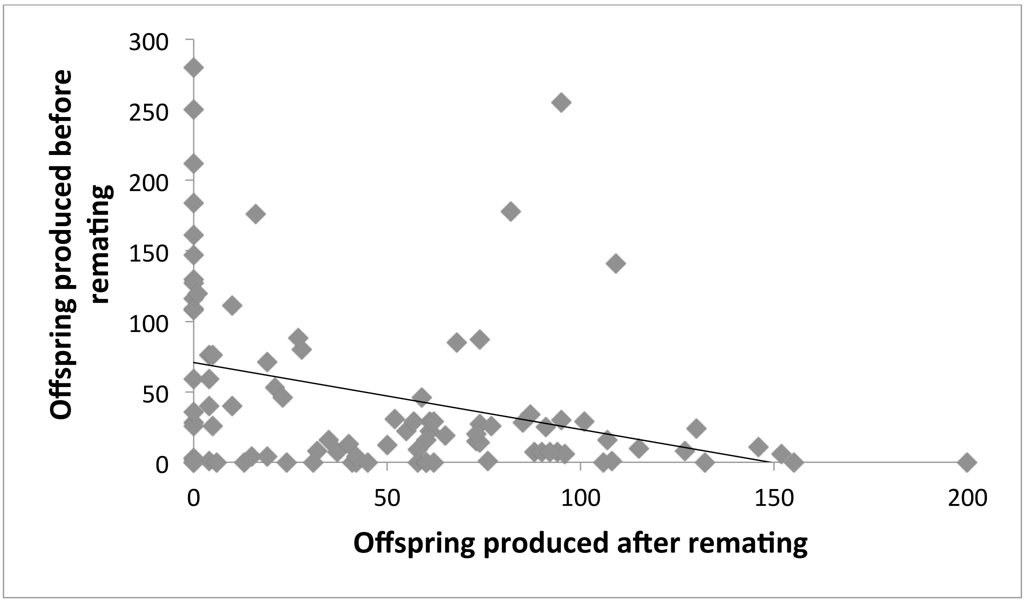

Figure S3 A 3D representation of the fitness surface for wild type experiment males for two post-mating traits: male induced length of remating interval and sperm defense ability. Relationships between the two traits and male fitness are shown (a) before and (b) after canonical rotation.

(a)

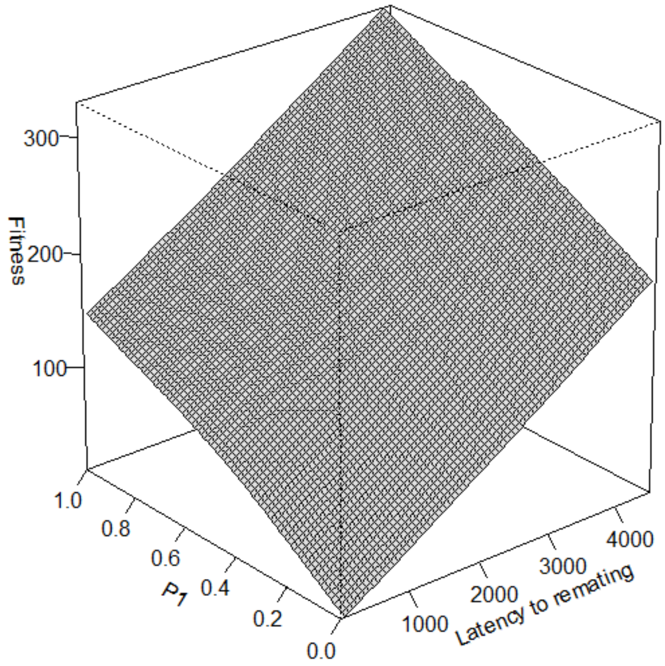

(b)

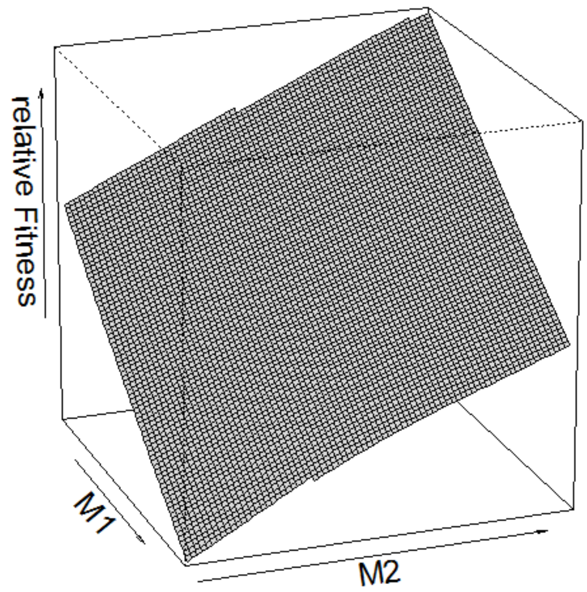

Figure S4 The relationship between the length of the intermating interval and the number of offspring produced during this interval for (a) *SP⁰* males and (b) *SP⁺* control males. The intermating interval, in minutes, was measured as time between the end of the first mating (with either a *SP⁰* or *SP⁺* male) and the start of a second mating (with a *Dah;Sb[1]* male).

(a)

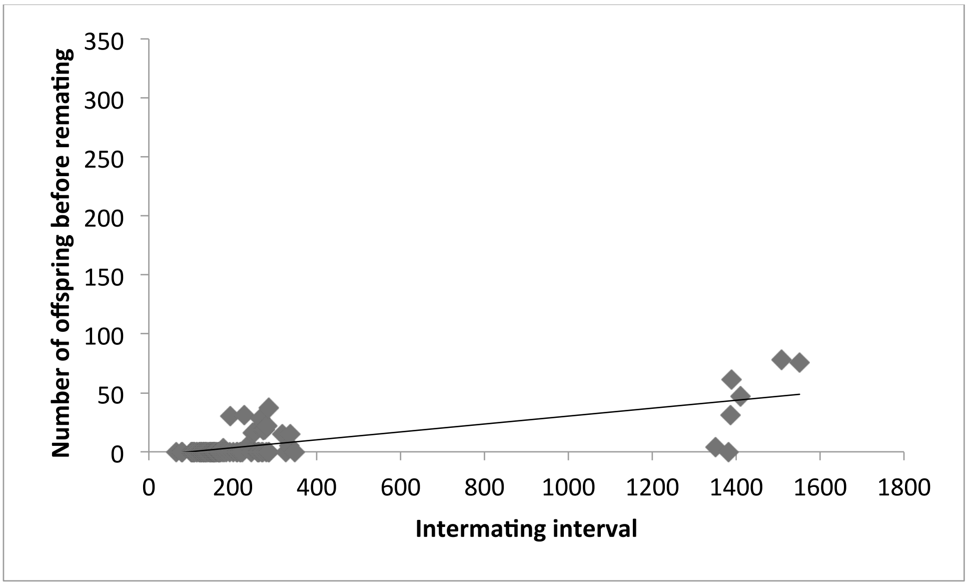

(b)

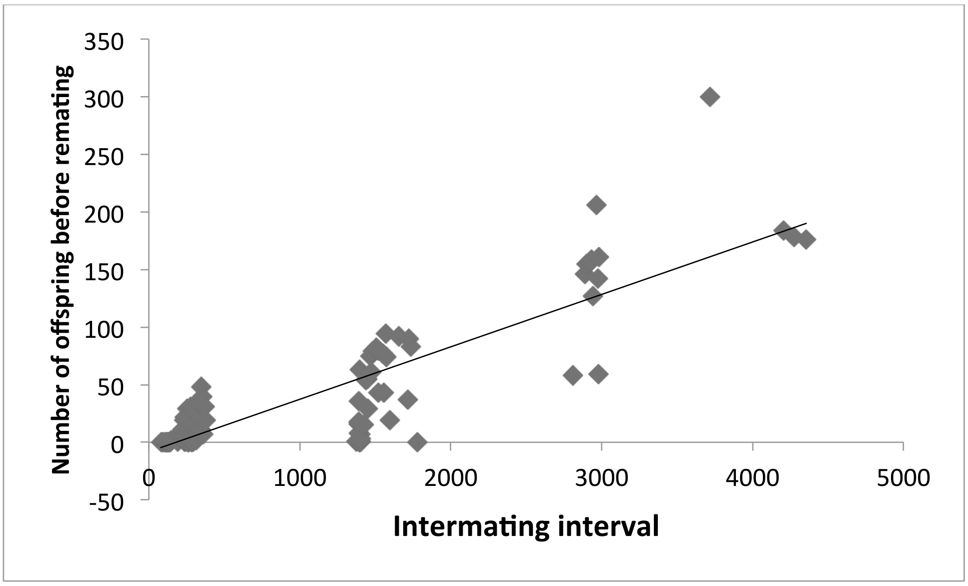

Figure S5 Fitness surfaces for SP^0 (a,c) and SP^+ (b,d) control males after canonical rotation, where axis M1 mainly represents the length of the remating interval and axis M2 a male's success in sperm defense. Panels (a) and (b) are 2D representations of fitness surfaces while (c) and (d) depict 3D images. The height and colour of the landscapes display the potential fitness gains resulting from the different combination of intermating interval (M1) and P1 (M2).

(a) SP^0

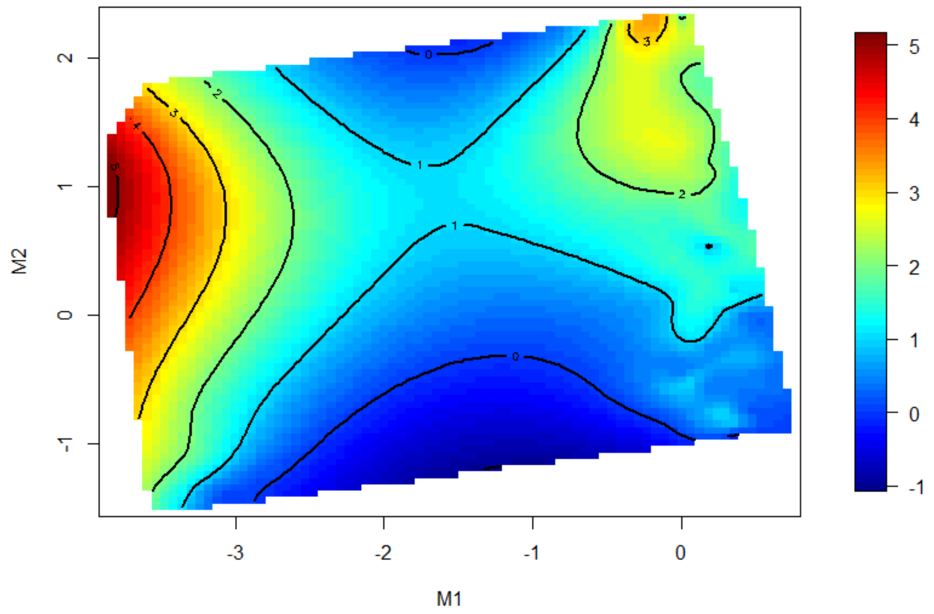

(b) SP^+

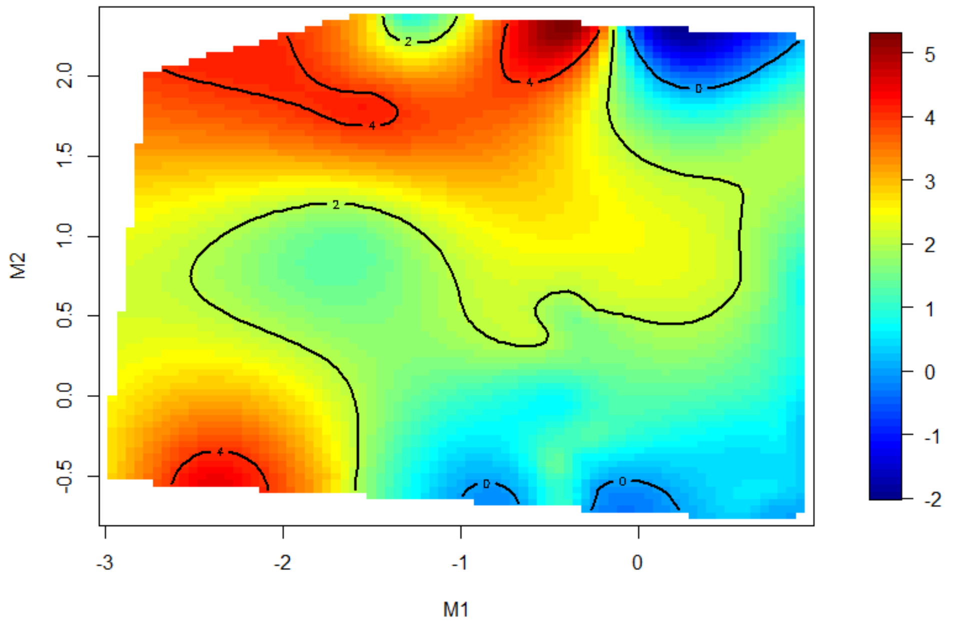

(c) *SP⁰*

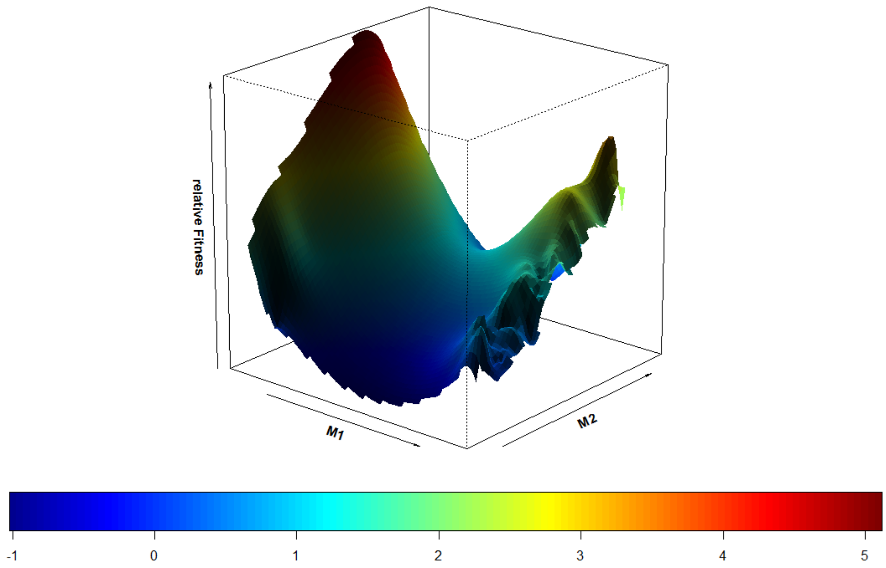
relative Fitness
M1
M2
-1
0
1
2
3
4
5

(d) *SP⁺*

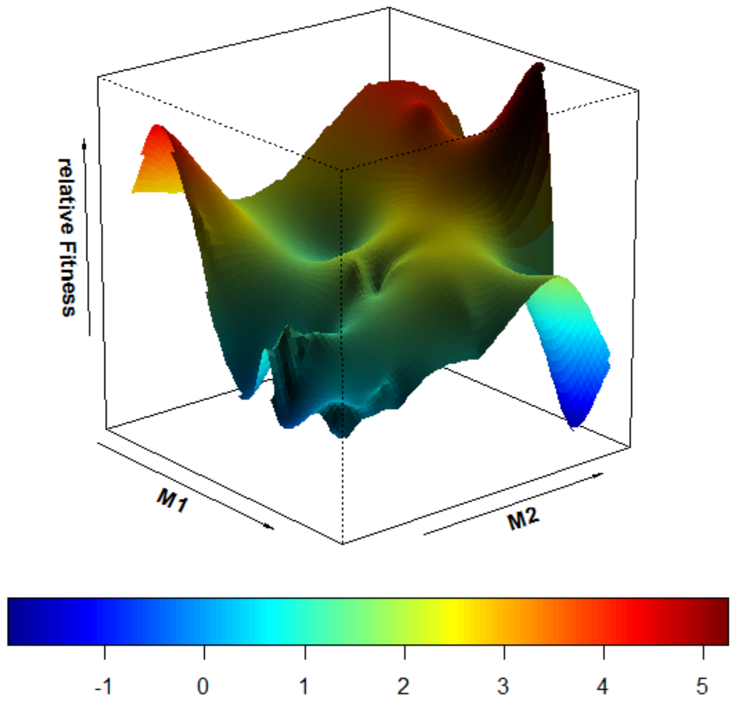
relative Fitness
M1
M2
-1
0
1
2
3
4
5

Reference

Stinchcombe, J.R., Agrawal, A.F., Hohenlohe, P.A., Arnold, S.J. & Blows, M.W. 2008. Estimating nonlinear selection gradients using quadratic regression coefficients: double or nothing? *Evolution* 62: 2435–2440.
